# A decade of experience with genetically tailored pig models for diabetes and metabolic research

**DOI:** 10.1590/1984-3143-AR2020-0064

**Published:** 2020-08-26

**Authors:** Silja Zettler, Simone Renner, Elisabeth Kemter, Arne Hinrichs, Nikolai Klymiuk, Mattias Backman, Evamaria Olga Riedel, Christiane Mueller, Elisabeth Streckel, Christina Braun-Reichhart, Ana Sofia Martins, Mayuko Kurome, Barbara Keßler, Valeri Zakhartchenko, Florian Flenkenthaler, Georg Josef Arnold, Thomas Fröhlich, Helmut Blum, Andreas Blutke, Rüdiger Wanke, Eckhard Wolf

**Affiliations:** 1 Molecular Animal Breeding and Biotechnology, Gene Center and Department of Veterinary Sciences, LMU Munich, Munich, Germany; 2 Center for Innovative Medical Models, Department of Veterinary Sciences, LMU Munich, Oberschleißheim, Germany; 3 German Center for Diabetes Research, Neuherberg, Germany; 4 Laboratory for Functional Genome Analysis, Gene Center, LMU Munich, Munich; 5 Research Unit Analytical Pathology, Helmholtz Zentrum München, German Research Center for Environmental Health, Neuherberg, Germany; 6 Institute of Veterinary Pathology, Center for Clinical Veterinary Medicine, LMU Munich, Munich, Germany

**Keywords:** pig model, diabetes, biobank, xenotransplantation

## Abstract

The global prevalence of diabetes mellitus and other metabolic diseases is rapidly increasing. Animal models play pivotal roles in unravelling disease mechanisms and developing and testing therapeutic strategies. Rodents are the most widely used animal models but may have limitations in their resemblance to human disease mechanisms and phenotypes. Findings in rodent models are consequently often difficult to extrapolate to human clinical trials. To overcome this ‘translational gap’, we and other groups are developing porcine disease models. Pigs share many anatomical and physiological traits with humans and thus hold great promise as translational animal models. Importantly, the toolbox for genetic engineering of pigs is rapidly expanding. Human disease mechanisms and targets can therefore be reproduced in pigs on a molecular level, resulting in precise and predictive porcine (PPP) models. In this short review, we summarize our work on the development of genetically (pre)diabetic pig models and how they have been used to study disease mechanisms and test therapeutic strategies. This includes the generation of reporter pigs for studying beta-cell maturation and physiology. Furthermore, genetically engineered pigs are promising donors of pancreatic islets for xenotransplantation. In summary, genetically tailored pig models have become an important link in the chain of translational diabetes and metabolic research.

## Introduction

Diabetes mellitus (DM), which is characterized by chronic hyperglycemia, has a high prevalence worldwide. In 2019, the International Diabetes Federation (IDF, 2019) estimated that there are 463 million DM cases worldwide in the age group 20-79 years. DM is estimated to increase to 700 million in 2045 (IDF, 2019). DM is a heterogeneous disease that has been categorized by the American Diabetes Association into four types:

- Type 1 diabetes (T1D), characterized by irreversible autoimmune destruction of insulin-producing beta cells;
- Type 2 diabetes (T2D), the most prevalent form of DM, often associated with obesity and characterized by insulin resistance and relative insulin deficiency;
- Gestational diabetes (GD), which usually occurs after 24 weeks of pregnancy;
- Other specific causes of diabetes.

Recently, refined classification systems have been introduced to cover sub-phenotypes of prediabetes and of adult onset diabetes and to facilitate more personalized diabetes treatments (reviewed in Renner et al., 2020).

The progressive course of most categories of diabetes cannot be stopped with current treatment options and carries a high risk of secondary alterations, such as diabetic nephropathy, neuropathy, retinopathy and cardiovascular complications.

**Figure 1:**
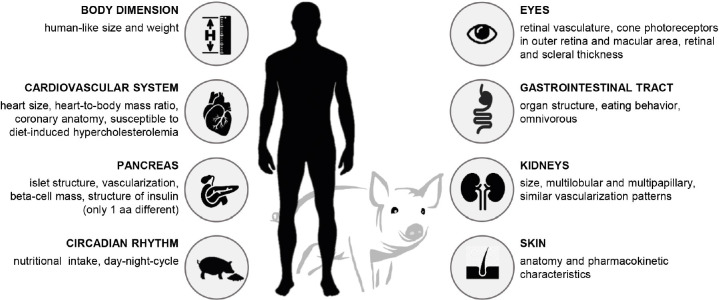
Anatomical and physiological similarities of pigs with humans.

Translational diabetes research is dedicated to improving and developing new diagnostic and therapeutic concepts. In this context, pigs are promising animal models due to many anatomical and physiological similarities with humans (Figure 1; reviewed in Aigner et al., 2010; Renner et al., 2016b). Like humans, pigs are omnivores and their gastrointestinal tract (GIT) is comparable to the human GIT. Porcine insulin differs from human insulin in only one amino acid. The insulin-producing beta cells as well as the overall structure of the pancreas (islets and vascularization) show many similarities to the human pancreas (reviewed in Hoang et al., 2014; Bakhti et al., 2019; Kim et al., 2020; Renner et al., 2020). There is a high degree of agreement with the human cardiovascular system (reviewed in Clauss et al., 2019) and kidneys (reviewed in Renner et al., 2020), which is why pigs are considered as promising donors for organ xenotransplantation (Längin et al., 2018; reviewed in Wolf et al., 2019). Moreover, the anatomy of the porcine skin (reviewed in Schneider and Wolf, 2016) and the structure of their eyes (Kleinwort et al., 2017) are similar to the human organs. In comparison to other large animal species used in diabetes research, such as dogs and non-human primates (reviewed in Kleinert et al., 2018; Ludwig et al., 2020), pigs have the advantage of better ethical acceptance, relatively low-cost reproduction and maintenance, and the existence of well-established methods for genetic modification (reviewed in Renner et al., 2016b). Finally, the body size of this species provides sufficient amounts of tissue and blood samples to easily carry out downstream experiments and analyses.

Traditionally, diabetic pig models have been generated by partial or total pancreatectomy, or by chemical destruction of the pancreatic beta cells using streptozotocin or alloxan. The advantages and problems of these treatments have been extensively reviewed recently (Renner et al., 2020). Feeding pigs with high-fat/high-energy diet results in obesity and insulin resistance (Renner et al., 2018), but rarely in clinical diabetes without further interventions. Combinations of several techniques are thus being used to mimic the multifactorial pathogenesis of T2D (reviewed in Renner et al., 2016b, 2020).

Genetic engineering provides important additional means for generating pig models for diabetes and metabolic research (Wolf et al., 2014). Over the past decades, the toolbox for genetic engineering of pigs has been continuously expanded (reviewed in Dmochewitz and Wolf, 2015; Whitelaw et al., 2016). Our lab has generated genetically (pre)diabetic pig models, but also transgenic pigs expressing reporter genes in islet cells for developmental studies of the endocrine pancreas or the optimization of islet culture. The latter is important for the clinical development of porcine islet xenotransplantation to restore beta-cell function in T1D patients (reviewed in Klymiuk et al., 2016; Kemter and Wolf, 2018). For free islet transplantation, the donor pigs are genetically modified to reduce or prevent their rejection by the recipients’ immune system (reviewed in Kemter et al., 2018).

In this short review, we summarize our own experiences with genetically engineered pigs used as models for diabetes and metabolic research or as donors for islet xenotransplantation. More comprehensive reviews on these topics have been published recently (Kemter et al., 2018; Kleinert et al., 2018; Renner et al., 2020). In addition, there are interesting attempts to generate human tissues in animal hosts (Wu et al., 2017), which have been discussed elsewhere (Wu et al., 2016a,b; Suchy and Nakauchi, 2018; Suchy et al., 2018).

## The GIPR^dn^ transgenic pig as a model for prediabetes

**Figure 2:**
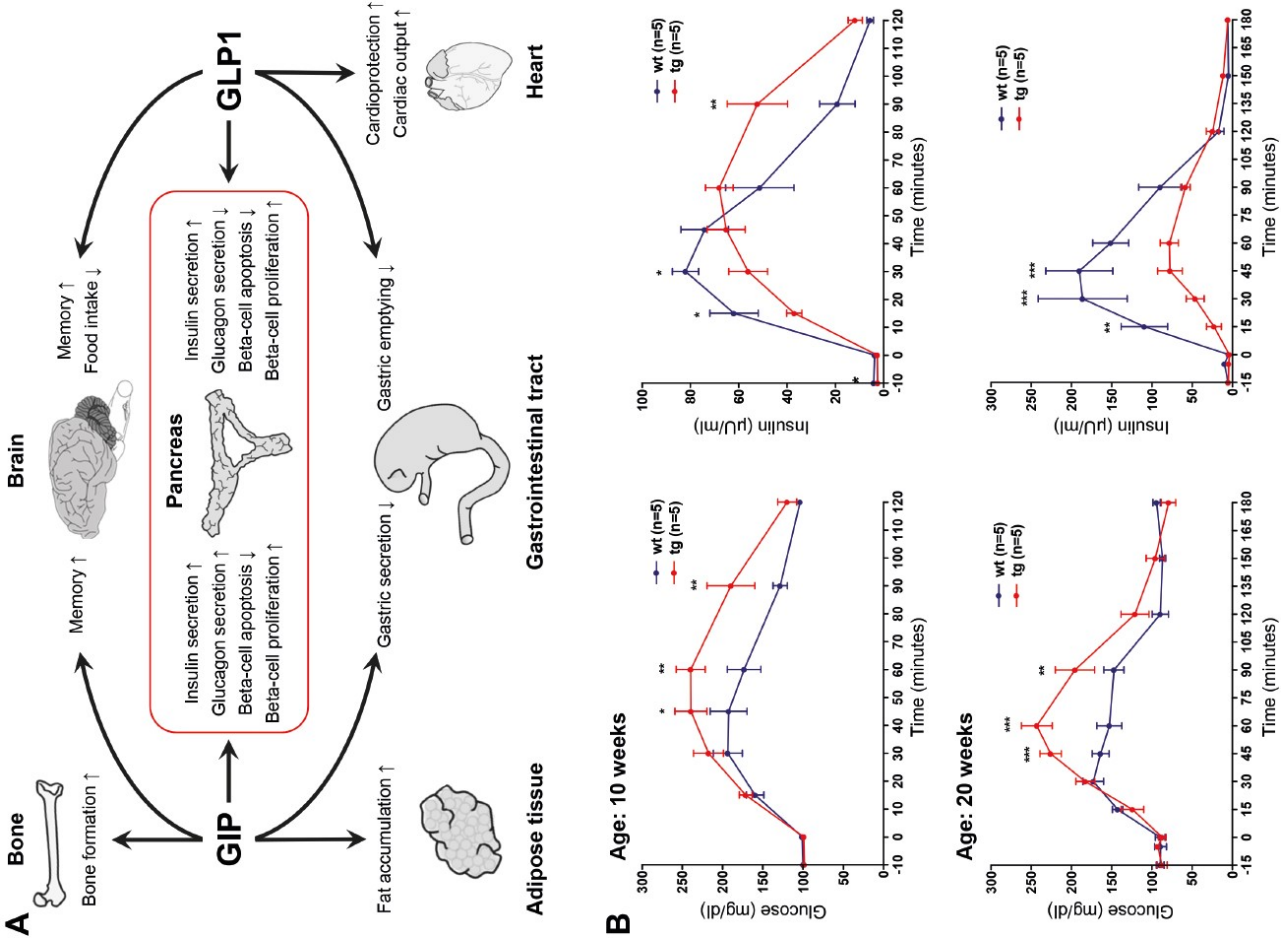
(A) Actions of the incretion hormones GIP and GLP1 on different organs; (B) Progressive deterioration of oral glucose tolerance in prediabetic GIPR^dn^ transgenic (tg) pigs compared to wild-type (wt) pigs (from Renner et al., 2010).

Insulin secretion is much greater in response to oral glucose compared to the same quantity of glucose administered intravenously. This phenomenon is attributed to the so-called ‘incretin effect’. The incretin hormones glucose-dependent insulinotropic polypeptide (GIP) and glucagon-like peptide-1 (GLP1) are secreted upon nutrient ingestion by specific endocrine cells in the small intestine. Among other functions, incretins bind to specific receptors of beta cells and potentiate insulin secretion (Figure 2A; reviewed in Renner et al., 2016a). This incretin effect is reduced in T2D patients as a consequence of impaired GIP function. To mimic this condition in a large animal model, we generated transgenic pigs expressing a dominant-negative GIP receptor (GIPR^dn^) under the control of a rat *Ins2* promoter sequence (Renner et al., 2010). These pigs were generated by injecting lentiviral vectors into the perivitelline space of zygotes (Hofmann et al., 2003). GIPR^dn^ binds GIP with similar affinity as the intact GIPR (a classical seven transmembrane domain G-protein coupled receptor), but has a deletion of 8 amino acids and an additional amino acid exchange in the third intracellular domain abolishing its signaling capacity (Renner et al., 2016a). The use of a rat *Ins2* promoter sequence facilitates expression of GIPR^dn^ in the beta cells. The GIPR^dn^ transgenic pig model resembles important aspects of prediabetes, including a reduced incretin effect, impaired glucose tolerance, initially delayed and in later stages quantitatively reduced insulin secretion (Figure 2B), and a progressive reduction of beta-cell mass (Renner et al., 2010). The reproducible and progressive phenotype of the GIPR^dn^ transgenic pig model was used in a targeted metabolomics approach to identify biomarker candidates, which exhibit changes in plasma concentration that correlate with the progression of the phenotype in the prediabetic period. In particular, it was possible to identify metabolomic signatures of amino acids and lipids that showed a high correlation with beta-cell mass (Renner et al., 2012). Moreover, the GIPR^dn^ transgenic pig model was used to characterize the effects of the GLP1 receptor agonist liraglutide, which is clinically approved for treatment of adult type 2 diabetics, in juvenile organisms. Specifically, it was possible to clarify whether the impaired function of the GIP/GIPR axis can be compensated by additional stimulation of the GLP1 receptor. Treatment of adolescent GIPR^dn^ transgenic pigs with liraglutide resulted in marked decreases in food intake and body weight gain and led to reduced postprandial circulating glucose and insulin levels compared to placebo-treated GIPR^dn^ transgenic pigs. This was probably due to an inhibitory effect of liraglutide on gastric emptying. Total alpha- and beta-cell mass was reduced in the liraglutide-treated group, but not when normalized for body weight. The 90-day liraglutide treatment had no effect on beta-cell proliferation nor on acinus-cell proliferation (Streckel et al., 2015).

## The *INS* ^C94Y^ transgenic pig: a clinically diabetic large animal model

**Figure 3:**
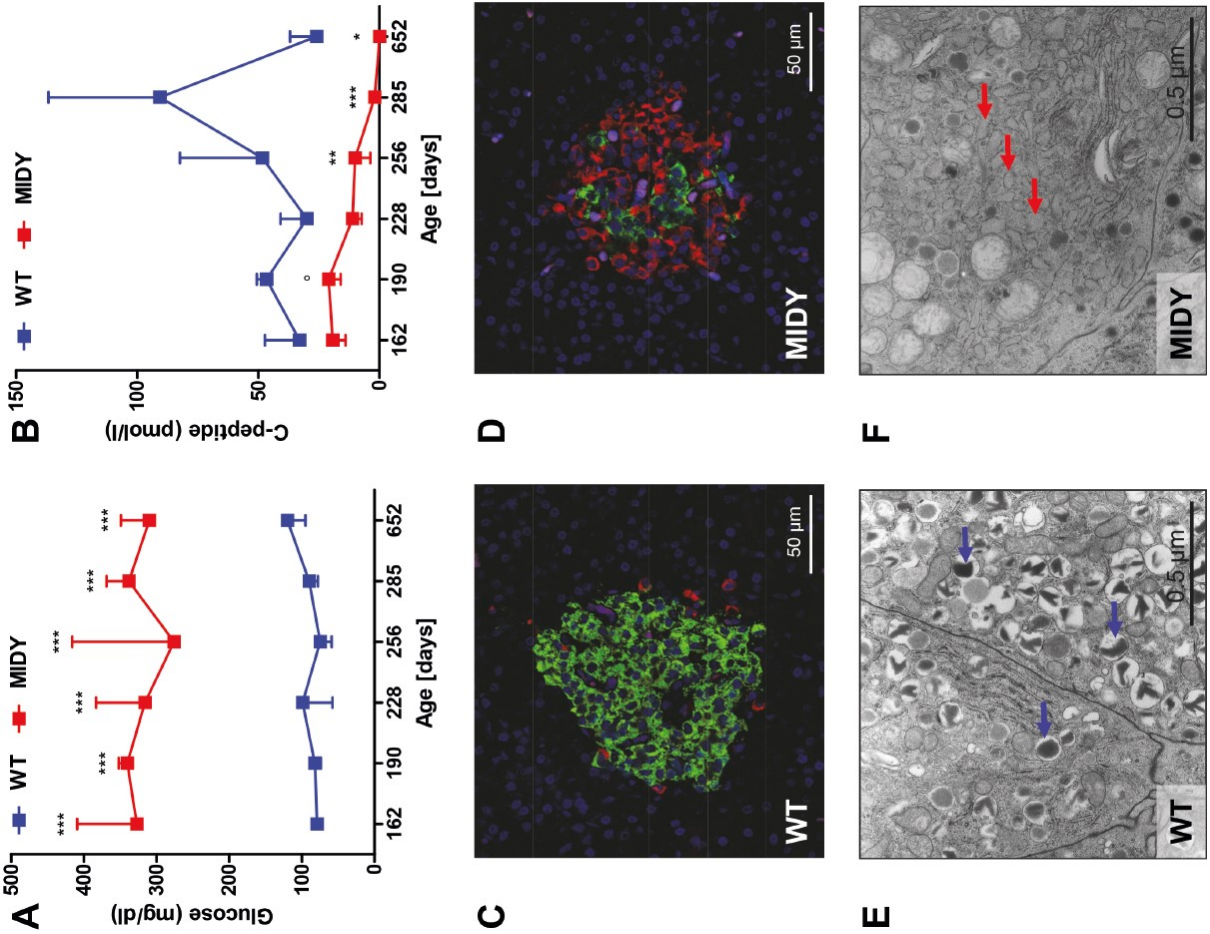
Consequences of expression of mutant insulin C94Y in mutant *INS* gene induced diabetes of youth (MIDY) pigs. (A) Permanently elevated fasting blood glucose levels; (B) Decreasing plasma C-peptide concentrations indicating perturbed insulin secretion and a decrease in beta-cell mass; (C, D) Immunofluorescence staining of pancreas sections for insulin (green) and glucagon (red). In islets of wild-type (WT) pigs, the majority of islet cells are beta cells (C), whereas in MIDY pigs the majority of beta cells are lost and alpha cells dominate (D; from Blutke et al., 2017); (E, F) Ultrastructural changes of beta cells from MIDY pigs (age: 4.5 months), which are indicative of ER stress. Beta cells of WT pigs (E) show multiple insulin granules (blue arrows). In beta cells from MIDY pigs, the number of insulin granules is markedly reduced and characteristic dilations of the endoplasmic reticulum are visible (E; red arrows; from Renner et al., 2013). *p < 0.05; **p < 0.01; ***p < 0.001.

The expression of mutant insulin may – depending on the type of mutation and the expression level – lead to permanent neonatal DM (now termed mutant *INS* gene induced diabetes of youth – MIDY, also known as maturity-onset diabetes of the young 10 – MODY10). In humans more than 50 different mutations of the *INS* gene are known (reviewed in Renner et al., 2016b). We generated transgenic pigs that express mutant insulin C94Y (Renner et al., 2013). A corresponding mutation was also found in MIDY patients. The MIDY pigs were generated by random insertion of an expression cassette including the porcine *INS* gene with the Cys→Tyr exchange at amino acid position 94 and essential regulatory elements into porcine fetal fibroblast cells. Pools of stable transfected cell clones were used for somatic cell nuclear transfer (SCNT) to produce transgenic founder piglets, and the line with the highest *INS* ^C94Y^ transgene expression was selected for further experiments (Renner et al., 2013). The C94Y mutation in our pig model disrupts one of the two disulfide bonds between the A and B chains of the insulin molecule, resulting in misfolded insulin, accumulation of proinsulin in the endoplasmic reticulum (ER), and chronic ER stress that cannot be solved by intrinsic repair mechanisms (the so-called “unfolded protein response” (UPR)). The cumulative effect results in beta-cell apoptosis. MIDY piglets get diabetic within the first week after birth. Since beta-cell mass is unaltered at this stage, a deficit in insulin secretion seems to be the primary cause. With increasing age, a loss of beta-cell mass is observed. At age 4.5 months, the beta-cell mass of MIDY pigs is 70% reduced compared to wild-type (WT) littermates and the beta cells show morphological hallmarks of ER stress (Figure 3). We thus developed an insulin substitution therapy, which resulted in restored normoglycemia and almost normal growth (Renner et al., 2013).

MIDY pigs are an interesting model for a broad range of applications, such as the preclinical testing of novel treatments or diagnostics (e.g. new insulin formulations, continuous glucose monitoring systems, insulin pumps, artificial pancreas), or the evaluation of early stages of diabetic complications in the kidneys, eyes, or microvasculature. Already at an age of 5 months, reduced capillarization and pericyte investment was observed in myocardium of MIDY pigs compared to age-matched controls. After experimental induction of an ischemic lesion, the myocardium responded with increased fibrosis. Local gene therapy with thymosin B4 markedly improved capillarization and pericyte investment in WT pigs, but only to a lesser extent in MIDY pigs (Hinkel et al., 2017). These findings are clinically relevant since reduced capillarization and pericyte investment is also observed in myocardium from diabetic patients.

To study the consequences of insulin insufficiency and chronic hyperglycemia in a multi-organ, multi-omics approach, we established a comprehensive biobank from four 2-year-old MIDY pigs and five age-matched WT controls (Blutke et al., 2017). In this context, the first standardized protocol for systematic sampling and processing of a broad spectrum of organs and tissues from porcine biomedical models was established (Albl et al., 2016; Blutke and Wanke, 2018). The Munich MIDY Pig Biobank harbors more than 20,000 redundant samples of different body fluids and of ~50 different organs and tissues. Tissue samples were preserved to facilitate holistic molecular profiling studies (e.g. of transcriptome, proteome, lipidome, metabolome), transcript and protein localization studies, and qualitative and quantitative pathohistological investigations.

To study the molecular consequences of chronic insulin deficiency for the liver, we analyzed liver samples of MIDY and WT pigs by RNA sequencing, proteomics, and targeted metabolomics/lipidomics. Multi-omics analyses revealed increased activities in amino acid metabolism, oxidation of fatty acids, ketogenesis, and gluconeogenesis in the MIDY samples. In particular, the concentrations of the ketogenic enzyme 3-hydroxy-3-methylglutaryl-CoA synthase 2 (HMGCS2) and of retinol dehydrogenase 16 (RDH16), which catalyzes the first step in retinoic acid biogenesis, were highly increased. Elevated levels of retinoic acid, which stimulates the expression of the gluconeogenic enzyme phosphoenolpyruvate carboxykinase (PCK1), were measured in the MIDY samples. In contrast, pathways related to the extracellular matrix and inflammation/pathogen defense response were less active than in the WT samples. This first multi-omics study of a clinically relevant diabetic large animal model revealed molecular signatures and key drivers of functional alterations of the liver in insulin-deficient diabetes mellitus (Backman et al., 2019).

A recent proteome study of immune cells from MIDY and WT pigs found distinct differences, in particular a significantly increased abundance of myosin regulatory light chain 9 (MLC-2C), which affects cell contractility by regulating myosin ATPase activity, in the MIDY samples (Weigand et al., 2020).

Molecular profiling studies of other tissues/cell types are ongoing. Particularly interesting is the retina of 2-year-old MIDY pigs, which showed diabetes-associated alterations with some similarities to diabetic retinopathy in human patients (Kleinwort et al., 2017).

## *INS* ^C93S^ transgenic pigs developing a milder diabetic phenotype

In addition to the *INS* ^C94Y^ transgenic model, we developed with a similar strategy transgenic pig lines expressing *INS* ^C93S^, giving rise to misfolded insulin due to disruption of an intrachain disulfide bond within the A-chain. *INS* ^C93S^ transgenic pigs showed impaired glucose tolerance due to reduced insulin secretion and mild fasting hyperglycemia. The milder phenotype of *INS* ^C93S^ vs. *INS* ^C94Y^ transgenic pigs is in line with lower expression of the mutant *INS* transgene (Renner et al., 2019). During pregnancy, insulin sensitivity decreased in both *INS* ^C93S^ transgenic and WT sows, but only the WT sows were able to compensate for the increased insulin demand with sufficiently increased insulin production to maintain normoglycemia. In contrast, *INS* ^C93S^ transgenic sows showed little compensatory capacity and maintained the mild fasting hyperglycemic level throughout pregnancy. This facilitated the study of potential metabolic programming effects of mild maternal hyperglycemia in the offspring. To separate direct effects of transgene expression from maternal effects, only WT offspring were investigated. Compared to offspring from WT sows, neonatal WT offspring from *INS* ^C93S^ transgenic sows revealed impaired glucose tolerance and insulin resistance with females being more severely affected than males. In addition, distinct changes in amino acid and lipid metabolism were observed, indicating that even mild maternal hyperglycemia can have significant metabolic programming effects in neonatal offspring (Renner et al., 2019).

## Metabolic alterations in GHR deficient pigs

Loss-of-function mutations of the growth hormone receptor (*GHR*) gene cause the rare autosomal recessive hereditary disease Laron syndrome (LS), which is characterized by short stature, obesity, and transient juvenile hypoglycemia. Moreover, a reduced incidence of diabetes mellitus in LS patients compared to controls was reported (Guevara-Aguirre et al., 2011), making this condition an interesting model for diabetes and metabolic research. For studies of the underlying mechanisms, we developed GHR deficient (*GHR*-KO) pigs as a large animal model for the human LS (Hinrichs et al., 2018). Frameshift mutations in the *GHR* gene were introduced by CRISPR-Cas9 in porcine zygotes. *GHR*-KO pigs show important hallmarks of the human disease, including reduced levels of insulin-like growth factor 1 (IGF1) and IGF-binding protein 3 (IGFBP3), increased serum GH concentrations, postnatal growth retardation, juvenile hypoglycemia, and a progressive increase in total body fat (Hinrichs et al., 2018).

To study the consequences of lacking GH action in the liver, a central target organ of GH, we performed holistic proteome and targeted metabolome analyses of liver samples from 6-month-old *GHR*-KO and control pigs (Riedel et al., 2020). GHR deficiency resulted in an increased abundance of enzymes involved in amino acid degradation, in the urea cycle, and in the tricarboxylic acid cycle. A decreased ratio of long-chain acylcarnitines to free carnitine suggested reduced activity of carnitine palmitoyltransferase 1A and thus reduced mitochondrial import of fatty acids for beta-oxidation. Increased levels of short-chain acylcarnitines in the liver and in the circulation of *GHR*-KO pigs may result from impaired beta-oxidation of short-chain fatty acids or from increased degradation of specific amino acids. The concentration of mono-unsaturated glycerophosphocholines was significantly increased in the liver of *GHR*-KO pigs without morphological signs of steatosis. The abundances of several proteins functionally linked to non-alcoholic fatty liver disease (fetuin B, retinol binding protein 4, several mitochondrial proteins) were, however, increased. Moreover, GHR deficient liver samples revealed distinct changes in the methionine and glutathione metabolic pathways. In particular, a significantly increased level of glycine N-methyltransferase and increased concentrations of total and free glutathione were observed. Several proteins revealed a sex-related abundance difference in the control group but not in the *GHR*-KO group, providing new insights into the role of GH in the sex-related specification of liver functions (Riedel et al., 2020).

## Transgenic pigs expressing reporter genes in the pancreatic islets

**Figure 4:**
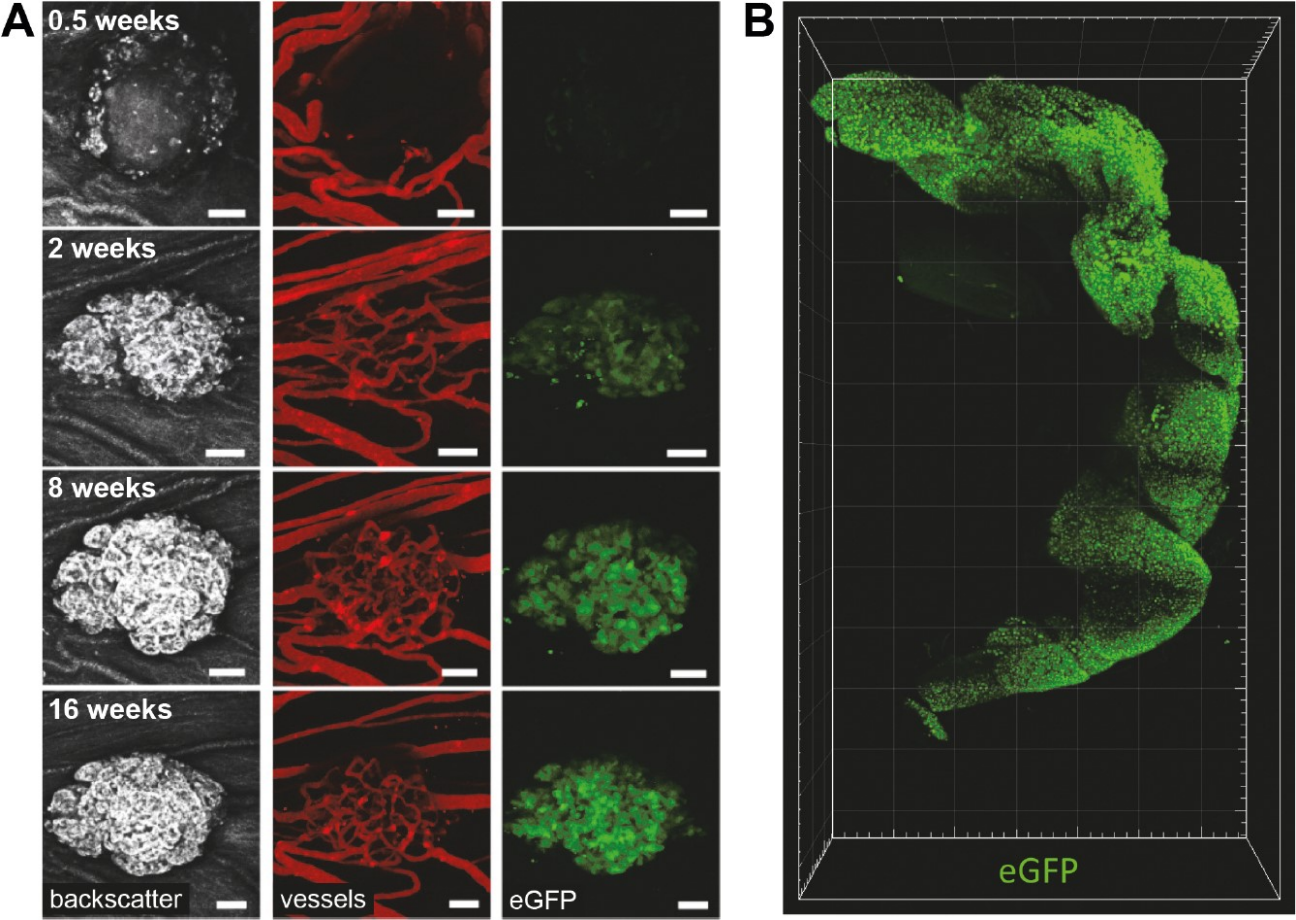
Neonatal islet-like cell clusters (NICCs) from *INS*-eGFP transgenic pigs can be used to study cell proliferation and maturation of NICCs *in vitro* and *in vivo*. (A) Maturation, expansion and vascularization of NICCs after transplantation into the anterior chamber of the mouse eye (from Kemter et al., 2017); (B) Visualization of pancreatic islets in total cleared porcine pancreas (from Zhao et al., 2020).

Functional studies of the endocrine cells in the islets of Langerhans are a prerequisite for better understanding the various forms of DM. Since access to human islets from healthy or diabetic subjects is limited, islets from rodent models are often used for *in vitro* studies. These rodent studies have revealed important knowledge on beta-cell function. Several therapeutic concepts for DM are based on the idea of generating additional beta cells, either via activating the regeneration of beta cells or by causing a transdifferentiation of pancreatic progenitor cells or other endocrine cell types into beta cells (reviewed in Zhou and Melton, 2018). Due to structural (e.g. distribution of the various endocrine cell types) and molecular differences (e.g. transcription factors of endocrine cells) between rodent and human islets of Langerhans, findings in rodent islets may not adequately resemble the situation in human islets. Alternatively, porcine islets of Langerhans can be used as a model. Islets of adult pigs are structurally similar to human islets (reviewed in Bakhti et al., 2019), but their isolation is difficult and expensive. In contrast, the isolation of neonatal islet-like cell clusters (NICCs) from piglets is less difficult, but NICCs are immature and require maturation *in vitro* (reviewed in Kemter and Wolf, 2018). To facilitate monitoring of this process in living cells, we generated transgenic pigs expressing enhanced green fluorescent protein (eGFP) under the control of the porcine *INS* promoter (Kemter et al., 2017). Transgenic pigs were generated by SCNT from *INS*-eGFP transfected cell clones. The use of this model facilitates *in vitro* and *in vivo* maturation studies of NICCs (Figure 4A) and molecular analyses of FACS-sorted beta cells (reviewed in Kemter et al., 2018). Recently, the distribution and structure of pancreatic islets *in situ* was revealed in total cleared pancreas from *INS*-eGFP transgenic pigs (Figure 4B; Zhao et al., 2020).

## Genetically modified pig islets for clinical xenotransplantation

T1D patients in very labile metabolic conditions are difficult to treat with insulin and may suffer from frequent episodes of severe unaware hypoglycemia. While islet allotransplantation would be the treatment of choice, human donor organs are limited. As a source for xenogeneic replacement of beta cells, either islets from adult donor pigs or NICCs may be used (reviewed in Klymiuk et al., 2016). Adult pig islets are difficult to isolate, and the donor pigs must be maintained under designated pathogen-free conditions for a long time. The isolation of NICCs is less problematic, but they are immature upon isolation and require time *in vitro* or *in vivo* to mature and become fully functional (reviewed in Kemter and Wolf, 2018). By using NICCs from *INS*-eGFP transgenic pigs this maturation process can be monitored *in vitro* (see above) and – after transplantation – *in vivo* (Cohrs et al., 2017; Kemter et al., 2017). To facilitate functional imaging studies using advanced modalities, such as multispectral optoacoustic tomography (MSOT) of transplanted islets or other tissues, we are in the process of generating and characterizing transgenic pigs that express near-infrared fluorescent protein (Dinnyes et al., 2020).

Rejection of xenografted porcine islets can be prevented by micro- or macroencapsulation (reviewed in Cooper et al., 2016; Klymiuk et al., 2016), or by genetic modification of the islet donor pigs (reviewed in Reichart et al., 2015; Kemter et al., 2018, 2020). The genetic modifications required depend on the transplantation site. Intraportal infusion of islets into the liver is so far the preferred application route, but intraperitoneal, subcutaneous, intramuscular islet delivery, and transplantation into the bone marrow have also been tested (reviewed in Kemter and Wolf, 2018).

**Figure 5:**
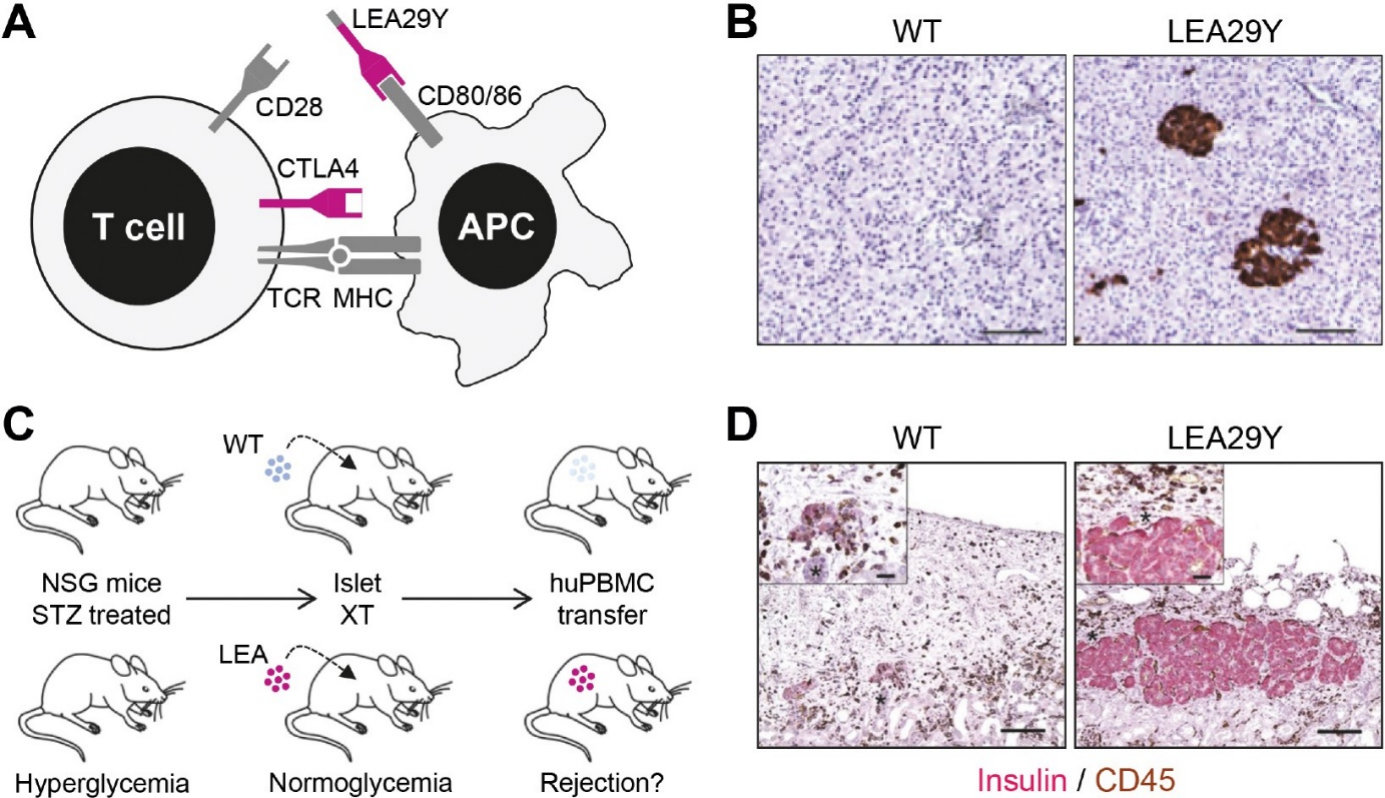
Protection of xenografted porcine pancreatic islets against T-cell mediated rejection by local expression of LEA29Y. (A) Principle of co-stimulation blockade of T cells. Activation of T cells requires interaction between the T-cell receptor (TCR) and a peptide-loaded major histocompatibility complex (MHC) on an antigen-presenting cell (APC). In addition, a second signal such as the interaction between CD28 und CD80/CD86 is required. The interaction of CTLA4 and CD80/CD86 blocks T-cell activation. The latter can also be achieved by the soluble molecule CTLA4-Ig or its affinity-optimized version LEA29Y; (B) Immunohistochemical staining of LEA29Y in pancreas sections; (C) Scheme for testing the efficacy of LEA29Y expression in xenotransplantation experiments of porcine islets into immunodeficient mice (NSG) transplanted with human immune cells (adapted from Wolf et al., 2019). Xenotransplantation (XT) of neonatal islet-like cell clusters (NICCs) from wild-type (WT) or *INS*-LEA29Y transgenic pigs (LEA29Y) into immunodeficient streptozotocin (STZ)-induced diabetic mice results in an insulin-positive cell mass that is able to normalize their blood glucose level. If the mice are subsequently reconstituted with human peripheral blood mononuclear cells (PBMCs), the WT islets are rejected while the LEA29Y transgenic islets are protected ((D); from Klymiuk et al., 2012). CD45 labels infiltrating T cells.

An important hurdle to successfully carry out clinical xenotransplantation of porcine islets is their T-cell mediated rejection. This can be overcome by preventing the co-stimulation of T cells. Activation of T cells involves the interaction of a T-cell receptor with a peptide-loaded MHC (major histocompatibility complex) molecule on an antigen-presenting cell (APC). In addition, a second signal that is induced by the interaction of co-stimulatory molecules on T cells and APCs is required. A prominent pair of co-stimulatory molecules is CD28 on T cells and CD80/CD86 on APCs (Figure 5A). Their interaction can be blocked by soluble molecules, such as CTLA4-Ig or LEA29Y, a variant with higher affinity for CD80/CD86, thus inhibiting T-cell activation. While these co-stimulation blocking agents are usually applied systemically, genetic engineering of the donor pigs facilitates their local expression in the xenograft, potentially preventing its T-cell mediated rejection without systemically blocking T-cell activation. To test this hypothesis for islet xenotransplantation, we generated by SCNT from transfected cells transgenic pigs expressing LEA29Y under the control of the porcine *INS* promoter specifically in the pancreatic beta cells (Figure 5B; Klymiuk et al., 2012). After transplantation into diabetic immunodeficient mice, LEA29Y transgenic and WT pig islets were able to restore glucose homeostasis. Subsequent application of human immune cells resulted in the rejection of the WT pig islets, whereas the LEA29Y transgenic islets were protected (Figure 5C, D). Interestingly, only very low concentrations of LEA29Y were detected in the circulation of the islet-grafted mice, supporting the concept of local suppression of T-cell mediated xenograft rejection (Klymiuk et al., 2012). A second study demonstrated that *INS*-LEA29Y transgenic pig islets can control glucose homeostasis of beta-cell deficient mice with a humanized immune system for many months (Wolf-van Buerck et al., 2017).

## Conclusions and perspectives

During the past decade, multiple genetically tailored pig models for diabetes and obesity research have been generated, characterized, and implemented in preclinical research projects (reviewed in Renner et al., 2020). Improvements in porcine whole genome resources (Walters et al., 2012) and molecular profiling techniques further increase the value of these translational animal models for addressing unresolved questions in diabetes research, such as the role of organ crosstalk in the development of diabetic complications. In this context, the integration of mouse, pig, and human data appears to be particularly promising (Vogel et al., 2018).

Pigs are, however, not only interesting disease models, but also promising donors of cells, tissues, and organs for xenotransplantation. Genetic modification of the donor pigs (reviewed in Kemter et al., 2018) can overcome the need for physical immune isolation (e.g. encapsulation) of transplanted islets and thus allow their immediate contact with the recipients’ blood vessels, ensuring that transplanted islets function in a manner that more closely mirrors standard human physiology. Guidelines for the clinical translation of porcine islet xenotransplantation have been established (Cowan et al., 2016). Genetically modified porcine islets are thus a realistic future option to overcome the lack of islet allotransplants for the treatment of T1D.

## References

[B001] Aigner B, Renner S, Kessler B, Klymiuk N, Kurome M, Wunsch A, Wolf E (2010). Transgenic pigs as models for translational biomedical research. J Mol Med.

[B002] Albl B, Haesner S, Braun-Reichhart C, Streckel E, Renner S, Seeliger F, Wolf E, Wanke R, Blutke A (2016). Tissue sampling guides for porcine biomedical models. Toxicol Pathol.

[B003] Backman M, Flenkenthaler F, Blutke A, Dahlhoff M, Ländström E, Renner S, Philippou-Massier J, Krebs S, Rathkolb B, Prehn C, Grzybek M, Coskun Ü, Rothe M, Adamski J, Angelis M, Wanke R, Fröhlich T, Arnold GJ, Blum H, Wolf E (2019). Multi-omics insights into functional alterations of the liver in insulin-deficient diabetes mellitus. Mol Metab.

[B004] Bakhti M, Böttcher A, Lickert H (2019). Modelling the endocrine pancreas in health and disease. Nat Rev Endocrinol.

[B005] Blutke A, Renner S, Flenkenthaler F, Backman M, Haesner S, Kemter E, Ländström E, Braun-Reichhart C, Albl B, Streckel E, Rathkolb B, Prehn C, Palladini A, Grzybek M, Krebs S, Bauersachs S, Bähr A, Brühschwein A, Deeg CA, De Monte E, Dmochewitz M, Eberle C, Emrich D, Fux R, Groth F, Gumbert S, Heitmann A, Hinrichs A, Keßler B, Kurome M, Leipig-Rudolph M, Matiasek K, Öztürk H, Otzdorff C, Reichenbach M, Reichenbach HD, Rieger A, Rieseberg B, Rosati M, Saucedo MN, Schleicher A, Schneider MR, Simmet K, Steinmetz J, Übel N, Zehetmaier P, Jung A, Adamski J, Coskun Ü, Angelis M, Simmet C, Ritzmann M, Meyer-Lindenberg A, Blum H, Arnold GJ, Fröhlich T, Wanke R, Wolf E (2017). The Munich MIDY Pig Biobank: a unique resource for studying organ crosstalk in diabetes. Mol Metab.

[B006] Blutke A, Wanke R (2018). Sampling strategies and processing of biobank tissue samples from porcine biomedical models. J Vis Exp.

[B007] Clauss S, Bleyer C, Schuttler D, Tomsits P, Renner S, Klymiuk N, Wakili R, Massberg S, Wolf E, Kaab S (2019). Animal models of arrhythmia: classic electrophysiology to genetically modified large animals. Nat Rev Cardiol.

[B008] Cohrs CM, Chen C, Jahn SR, Stertmann J, Chmelova H, Weitz J, Bahr A, Klymiuk N, Steffen A, Ludwig B, Kamvissi V, Wolf E, Bornstein SR, Solimena M, Speier S (2017). Vessel network architecture of adult human islets promotes distinct cell-cell interactions in situ and is altered after transplantation. Endocrinology.

[B009] Cooper DK, Matsumoto S, Abalovich A, Itoh T, Mourad NI, Gianello PR, Wolf E, Cozzi E (2016). Progress in clinical encapsulated islet xenotransplantation. Transplantation.

[B010] Cowan PJ, Ayares D, Wolf E, Cooper DK (2016). First update of the International Xenotransplantation Association consensus statement on conditions for undertaking clinical trials of porcine islet products in type 1 diabetes--Chapter 2b: genetically modified source pigs. Xenotransplantation.

[B011] Dinnyes A, Schnur A, Muenthaisong S, Bartenstein P, Burcez CT, Burton N, Cyran C, Gianello P, Kemter E, Nemeth G, Nicotra F, Prepost E, Qiu Y, Russo L, Wirth A, Wolf E, Ziegler S, Kobolak J (2020). Integration of nano- and biotechnology for beta-cell and islet transplantation in type-1 diabetes treatment. Cell Prolif.

[B012] Dmochewitz M, Wolf E (2015). Genetic engineering of pigs for the creation of translational models of human pathologies. Anim Front.

[B013] Guevara-Aguirre J, Balasubramanian P, Guevara-Aguirre M, Wei M, Madia F, Cheng CW, Hwang D, Martin-Montalvo A, Saavedra J, Ingles S, de Cabo R, Cohen P, Longo VD (2011). Growth hormone receptor deficiency is associated with a major reduction in pro-aging signaling, cancer, and diabetes in humans. Sci Transl Med.

[B014] Hinkel R, Howe A, Renner S, Ng J, Lee S, Klett K, Kaczmarek V, Moretti A, Laugwitz KL, Skroblin P, Mayr M, Milting H, Dendorfer A, Reichart B, Wolf E, Kupatt C (2017). Diabetes mellitus-induced microvascular destabilization in the myocardium. J Am Coll Cardiol.

[B015] Hinrichs A, Kessler B, Kurome M, Blutke A, Kemter E, Bernau M, Scholz AM, Rathkolb B, Renner S, Bultmann S, Leonhardt H, Angelis M, Nagashima H, Hoeflich A, Blum WF, Bidlingmaier M, Wanke R, Dahlhoff M, Wolf E (2018). Growth hormone receptor-deficient pigs resemble the pathophysiology of human Laron syndrome and reveal altered activation of signaling cascades in the liver. Mol Metab.

[B016] Hoang DT, Matsunari H, Nagaya M, Nagashima H, Millis JM, Witkowski P, Periwal V, Hara M, Jo J (2014). A conserved rule for pancreatic islet organization. PLoS One.

[B017] Hofmann A, Kessler B, Ewerling S, Weppert M, Vogg B, Ludwig H, Stojkovic M, Boelhauve M, Brem G, Wolf E, Pfeifer A (2003). Efficient transgenesis in farm animals by lentiviral vectors. EMBO Rep.

[B018] IDF (2019). IDF diabetes atlas.

[B019] Kemter E, Cohrs CM, Schafer M, Schuster M, Steinmeyer K, Wolf-van Buerck L, Wolf A, Wuensch A, Kurome M, Kessler B, Zakhartchenko V, Loehn M, Ivashchenko Y, Seissler J, Schulte AM, Speier S, Wolf E (2017). INS-eGFP transgenic pigs: a novel reporter system for studying maturation, growth and vascularisation of neonatal islet-like cell clusters. Diabetologia.

[B020] Kemter E, Denner J, Wolf E (2018). Will genetic engineering carry xenotransplantation of pig islets to the clinic?. Curr Diab Rep.

[B021] Kemter E, Schnieke A, Fischer K, Cowan PJ, Wolf E (2020). Xeno-organ donor pigs with multiple genetic modifications - the more the better?. Curr Opin Genet Dev.

[B022] Kemter E, Wolf E (2018). Recent progress in porcine islet isolation, culture and engraftment strategies for xenotransplantation. Curr Opin Organ Transplant.

[B023] Kim S, Whitener RL, Peiris H, Gu X, Chang CA, Lam JY, Camunas-Soler J, Park I, Bevacqua RJ, Tellez K, Quake SR, Lakey JRT, Bottino R, Ross PJ, Kim SK (2020). Molecular and genetic regulation of pig pancreatic islet cell development. Development.

[B024] Kleinert M, Clemmensen C, Hofmann SM, Moore MC, Renner S, Woods SC, Huypens P, Beckers J, Angelis M, Schurmann A, Bakhti M, Klingenspor M, Heiman M, Cherrington AD, Ristow M, Lickert H, Wolf E, Havel PJ, Muller TD, Tschop MH (2018). Animal models of obesity and diabetes mellitus. Nat Rev Endocrinol.

[B025] Kleinwort KJH, Amann B, Hauck SM, Hirmer S, Blutke A, Renner S, Uhl PB, Lutterberg K, Sekundo W, Wolf E, Deeg CA (2017). Retinopathy with central oedema in an INS (C94Y) transgenic pig model of long-term diabetes. Diabetologia.

[B026] Klymiuk N, Ludwig B, Seissler J, Reichart B, Wolf E (2016). Current concepts of using pigs as a source for beta-cell replacement therapy of type 1 diabetes. Curr Mol Biol Rep.

[B027] Klymiuk N, van Buerck L, Bahr A, Offers M, Kessler B, Wuensch A, Kurome M, Thormann M, Lochner K, Nagashima H, Herbach N, Wanke R, Seissler J, Wolf E (2012). Xenografted islet cell clusters from INSLEA29Y transgenic pigs rescue diabetes and prevent immune rejection in humanized mice. Diabetes.

[B028] Längin M, Mayr T, Reichart B, Michel S, Buchholz S, Guethoff S, Dashkevich A, Baehr A, Egerer S, Bauer A, Mihalj M, Panelli A, Issl L, Ying J, Fresch AK, Buttgereit I, Mokelke M, Radan J, Werner F, Lutzmann I, Steen S, Sjoberg T, Paskevicius A, Qiuming L, Sfriso R, Rieben R, Dahlhoff M, Kessler B, Kemter E, Kurome M, Zakhartchenko V, Klett K, Hinkel R, Kupatt C, Falkenau A, Reu S, Ellgass R, Herzog R, Binder U, Wich G, Skerra A, Ayares D, Kind A, Schonmann U, Kaup FJ, Hagl C, Wolf E, Klymiuk N, Brenner P, Abicht JM (2018). Consistent success in life-supporting porcine cardiac xenotransplantation. Nature.

[B029] Ludwig B, Wolf E, Schonmann U, Ludwig S (2020). Large animal models of diabetes. Methods Mol Biol.

[B030] Reichart B, Niemann H, Chavakis T, Denner J, Jaeckel E, Ludwig B, Marckmann G, Schnieke A, Schwinzer R, Seissler J, Tonjes RR, Klymiuk N, Wolf E, Bornstein SR (2015). Xenotransplantation of porcine islet cells as a potential option for the treatment of type 1 diabetes in the future. Horm Metab Res.

[B031] Renner S, Blutke A, Clauss S, Deeg CA, Kemter E, Merkus D, Wanke R, Wolf E (2020). Porcine models for studying complications and organ crosstalk in diabetes mellitus. Cell Tissue Res.

[B032] Renner S, Blutke A, Dobenecker B, Dhom G, Muller TD, Finan B, Clemmensen C, Bernau M, Novak I, Rathkolb B, Senf S, Zols S, Roth M, Gotz A, Hofmann SM, Angelis M, Wanke R, Kienzle E, Scholz AM, DiMarchi R, Ritzmann M, Tschop MH, Wolf E (2018). Metabolic syndrome and extensive adipose tissue inflammation in morbidly obese Gottingen minipigs. Mol Metab.

[B033] Renner S, Blutke A, Streckel E, Wanke R, Wolf E (2016). Incretin actions and consequences of incretin-based therapies: lessons from complementary animal models. J Pathol.

[B034] Renner S, Braun-Reichhart C, Blutke A, Herbach N, Emrich D, Streckel E, Wunsch A, Kessler B, Kurome M, Bahr A, Klymiuk N, Krebs S, Puk O, Nagashima H, Graw J, Blum H, Wanke R, Wolf E (2013). Permanent neonatal diabetes in INS(C94Y) transgenic pigs. Diabetes.

[B035] Renner S, Dobenecker B, Blutke A, Zols S, Wanke R, Ritzmann M, Wolf E (2016). Comparative aspects of rodent and nonrodent animal models for mechanistic and translational diabetes research. Theriogenology.

[B036] Renner S, Fehlings C, Herbach N, Hofmann A, von Waldthausen DC, Kessler B, Ulrichs K, Chodnevskaja I, Moskalenko V, Amselgruber W, Goke B, Pfeifer A, Wanke R, Wolf E (2010). Glucose intolerance and reduced proliferation of pancreatic beta-cells in transgenic pigs with impaired glucose-dependent insulinotropic polypeptide function. Diabetes.

[B037] Renner S, Martins AS, Streckel E, Braun-Reichhart C, Backman M, Prehn C, Klymiuk N, Bahr A, Blutke A, Landbrecht-Schessl C, Wunsch A, Kessler B, Kurome M, Hinrichs A, Koopmans SJ, Krebs S, Kemter E, Rathkolb B, Nagashima H, Blum H, Ritzmann M, Wanke R, Aigner B, Adamski J, Angelis M, Wolf E (2019). Mild maternal hyperglycemia in INS (C93S) transgenic pigs causes impaired glucose tolerance and metabolic alterations in neonatal offspring. Dis Model Mech.

[B038] Renner S, Römisch-Margl W, Prehn C, Krebs S, Adamski J, Göke B, Blum H, Suhre K, Roscher AA, Wolf E (2012). Changing metabolic signatures of amino acids and lipids during the prediabetic period in a pig model with impaired incretin function and reduced β-cell mass. Diabetes.

[B039] Riedel EO, Hinrichs A, Kemter E, Dahlhoff M, Backman M, Rathkolb B, Prehn C, Adamski J, Renner S, Blutke A, Angelis M, Bidlingmaier M, Schopohl J, Arnold GJ, Frohlich T, Wolf E (2020). Functional changes of the liver in the absence of growth hormone (GH) action - proteomic and metabolomic insights from a GH receptor deficient pig model. Mol Metab.

[B040] Schneider MR, Wolf E (2016). Genetically engineered pigs as investigative and translational models in dermatology. Br J Dermatol.

[B041] Streckel E, Braun-Reichhart C, Herbach N, Dahlhoff M, Kessler B, Blutke A, Bahr A, Ubel N, Eddicks M, Ritzmann M, Krebs S, Goke B, Blum H, Wanke R, Wolf E, Renner S (2015). Effects of the glucagon-like peptide-1 receptor agonist liraglutide in juvenile transgenic pigs modeling a pre-diabetic condition. J Transl Med.

[B042] Suchy F, Nakauchi H (2018). Interspecies chimeras. Curr Opin Genet Dev.

[B043] Suchy F, Yamaguchi T, Nakauchi H (2018). iPSC-derived organs in vivo: challenges and promise. Cell Stem Cell.

[B044] Vogel H, Kamitz A, Hallahan N, Lebek S, Schallschmidt T, Jonas W, Jahnert M, Gottmann P, Zellner L, Kanzleiter T, Damen M, Altenhofen D, Burkhardt R, Renner S, Dahlhoff M, Wolf E, Muller TD, Bluher M, Joost HG, Chadt A, Al-Hasani H, Schurmann A (2018). A collective diabetes cross in combination with a computational framework to dissect the genetics of human obesity and Type 2 diabetes. Hum Mol Genet.

[B045] Walters EM, Wolf E, Whyte JJ, Mao J, Renner S, Nagashima H, Kobayashi E, Zhao J, Wells KD, Critser JK, Riley LK, Prather RS (2012). Completion of the swine genome will simplify the production of swine as a large animal biomedical model. BMC Med Genomics.

[B046] Weigand M, Degroote RL, Amann B, Renner S, Wolf E, Hauck SM, Deeg CA (2020). Proteome profile of neutrophils from a transgenic diabetic pig model shows distinct changes. J Proteomics.

[B047] Whitelaw CB, Sheets TP, Lillico SG, Telugu BP (2016). Engineering large animal models of human disease. J Pathol.

[B048] Wolf E, Braun-Reichhart C, Streckel E, Renner S (2014). Genetically engineered pig models for diabetes research. Transgenic Res.

[B049] Wolf E, Kemter E, Klymiuk N, Reichart B (2019). Genetically modified pigs as donors of cells, tissues, and organs for xenotransplantation. Anim Front.

[B050] Wolf-van Buerck L, Schuster M, Oduncu FS, Baehr A, Mayr T, Guethoff S, Abicht J, Reichart B, Klymiuk N, Wolf E, Seissler J (2017). LEA29Y expression in transgenic neonatal porcine islet-like cluster promotes long-lasting xenograft survival in humanized mice without immunosuppressive therapy. Sci Rep.

[B051] Wu J, Greely HT, Jaenisch R, Nakauchi H, Rossant J, Belmonte JC (2016). Stem cells and interspecies chimaeras. Nature.

[B052] Wu J, Platero Luengo A, Gil MA, Suzuki K, Cuello C, Morales Valencia M, Parrilla I, Martinez CA, Nohalez A, Roca J, Martinez EA, Izpisua Belmonte JC (2016). Generation of human organs in pigs via interspecies blastocyst complementation. Reprod Domest Anim.

[B053] Wu J, Platero-Luengo A, Sakurai M, Sugawara A, Gil MA, Yamauchi T, Suzuki K, Bogliotti YS, Cuello C, Morales Valencia M, Okumura D, Luo J, Vilariño M, Parrilla I, Soto DA, Martinez CA, Hishida T, Sánchez-Bautista S, Martinez-Martinez ML, Wang H, Nohalez A, Aizawa E, Martinez-Redondo P, Ocampo A, Reddy P, Roca J, Maga EA, Esteban CR, Berggren WT, Nuñez Delicado E, Lajara J, Guillen I, Guillen P, Campistol JM, Martinez EA, Ross PJ, Izpisua Belmonte JC (2017). Interspecies chimerism with mammalian pluripotent stem cells. Cell.

[B054] Zhao S, Todorov MI, Cai R, Maskari RA, Steinke H, Kemter E, Mai H, Rong Z, Warmer M, Stanic K, Schoppe O, Paetzold JC, Gesierich B, Wong MN, Huber TB, Duering M, Bruns OT, Menze B, Lipfert J, Puelles VG, Wolf E, Bechmann I, Erturk A (2020). Cellular and molecular probing of intact human organs. Cell.

[B055] Zhou Q, Melton DA (2018). Pancreas regeneration. Nature.

